# Quantum phase transition induced by real-space topology

**DOI:** 10.1038/srep39416

**Published:** 2016-12-22

**Authors:** C. Li, G. Zhang, S. Lin, Z. Song

**Affiliations:** 1 School of Physics, Nankai University, Tianjin 300071, China; 2 College of Physics and Materials Science, Tianjin Normal University, Tianjin 300387, China

## Abstract

A quantum phase transition (QPT), including both topological and symmetry breaking types, is usually induced by the change of global parameters, such as external fields or global coupling constants. In this work, we demonstrate the existence of QPT induced by the real-space topology of the system. We investigate the groundstate properties of the tight-binding model on a honeycomb lattice with the torus geometry based on exact results. It is shown that the ground state experiences a second-order QPT, exhibiting the scaling behavior, when the torus switches to a tube, which reveals the connection between quantum phase and the real-space topology of the system.

Quantum phase transitions (QPTs) are of central interest both in the fields of condensed matter physics and quantum information. The transition describes an abrupt change in the ground state of a many-body system due to its quantum fluctuations. In general, a global physical parameter, such as external fields or widely distributed coupling constants may drive QPTs, including both topological^1^ and symmetry breaking types^2^. During the transition, the real-space geometry of the system is usually unchanged. A natural question is whether a change of real-space topology can induce a QPT for some peculiar cases. It is traced back to a problem in classical physics: the effect of a magnetic field on an object depends on its real-space topology (see the illustration in Fig. 1). A magnetic field affects a conducting loop via the magnetic flux, which is independent of its shape, for instance, no matter it is a metal donut or cup. However, it has no effect on a metal bar. The switch of the topology is equivalent to the sudden removal of the applied magnetic field, which may introduce a sudden change of the quantum state at zero temperature.

In this work, we demonstrate the influence of the real-space topology to the quantum phase via concrete tight-binding models on a honeycomb and square lattices, respectively. The change of the topology is presented by the value of hopping constants, which connecting the two ends of a tube. When the boundary hopping constants vary from finite to zero, a torus switches to a tube. We investigate the groundstate property as the function of the boundary coupling. Analytical and numerical results show that the ground state exhibits the scaling behavior of second-order QPTs in the honeycomb lattice, but not in the square lattice. It reveals that a geometric topological transition may induce a QPT in certain systems, which display physics beyond the current understanding of the QPT. The possible relation between QPTs and the geometric quantity in real-space may open attractive topics for different scientific communities.

## Results

### Graphene torus

**Figure 2:**
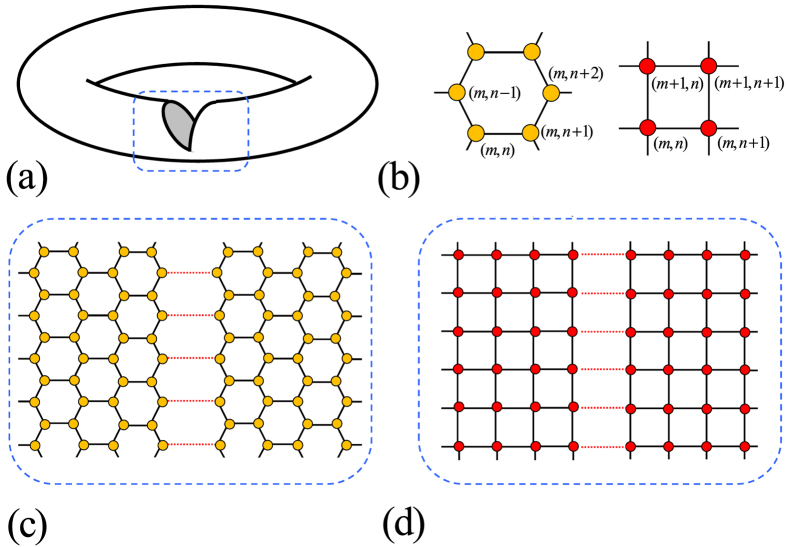
Schematic illustration of the lattice systems, which are employed to construct the systems (**a**) with different real-space topologies. We consider two types of lattices: (**c**) A honeycomb lattice; (**d**) A square lattice. A cut or broken torus is presented by the weak or zero hopping constants, which are indicated by dashed lines. The site index of two types of lattices is indicated in (**b**).

We consider a system of noninteracting particles in a honeycomb geometry, subjected to a magnetic flux *ϕ*. The tight-binding model for this system can be described by the Hamiltonian


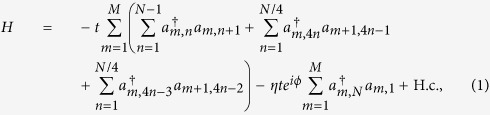


where *a*_*m,n*_ (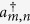) annihilates (creates) an electron in site (*m, n*) on an *M* × *N* lattice with integer *N*/4 and 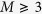, and obeys the periodic boundary conditions, *a*_*M* + 1,4*n*−1_ = *a*_1,4*n*−1_ and *a*_*M* + 1,4*n*−2_ = *a*_1,4*n*−2_, with *m* ∈ [1, *M*], *n* ∈ [1, *N*/4]. Parameter *t* is hopping integral. Here *ϕ* = 2*π*Φ/Φ_0_, where Φ is the flux threading the ring, Φ_0_ is the flux quantum. In Fig. 2, the geometry of the model is illustrated schematically. In this model, factor *η* is important, determining the boundary condition of the system. In the view of geometry, the value of *η* measures the topology of the system: nonzero *η* corresponds to a torus whereas zero *η* stands for a bar. The aim of this work is to explore what happens to the ground state when *η* passes the zero point. To this end, exact result is preferable. We note that the value of *η* does not affect the translational symmetry in another direction, so we employ the Fourier transformation


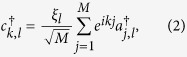


to rewrite the Hamiltonian, where *ξ*_*l*_ = 1 for *l* = 4*n*, 4*n* − 3 and *ξ*_*l*_ = *e*^−*ik*/2^ for *l* = 4*n* − 1, 4*n* − 2 with *n* ∈ [1, *N*/4], and *k* = 2*πm/M, m* ∈ [1, *M*]. The Hamiltonian can be expressed as *H* = ∑_*k*_*H*_*k*_, where


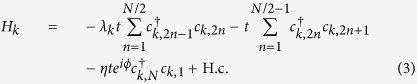


Together with [*H*_*k*_, *H*_*k*′_] = 0, we find that *H* is a combination of *M* independent Peierls rings with the *k*-dependent hopping integral *λ*_*k*_ = 2cos(*k*/2).

The one-dimensional dimerized Peierls system at half-filling, proposed by Su, Schrieffer, and Heeger (SSH) to model polyacetylene^3^,^4^, is the prototype of a topologically nontrivial band insulator with a symmetry protected topological (SPT) phase^5^,^6^. In recent years, extensive studies have been received^1^,^7^,^8^,^9^,^10^. For the open boundary condition, the number of zero modes reflects the winding number as a topological invariant, according to the bulk-boundary correspondence. Specifically, when *η* = 0, and infinite *N*, there are two zero modes if |*λ*_*k*_| < 1, but not if |*λ*_*k*_| > 1. Accordingly, there are approximately *M*/3 pairs of zero modes for a graphene tube with the open boundary condition. It indicates that the groundstate energy changes arising from the formation of the zero modes. We are interested in this process.

According to the Methods, we know that there is a pair of approximate solution for the two zero modes of Eq. (3) for small *η*, which are


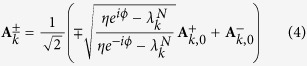


with eigenvalues


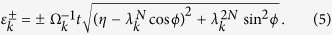


Where


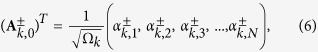


with 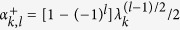, 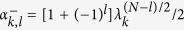 and 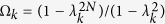

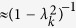
. These analytical expressions are the base for the investigation of the quantum phase transition induced by *η*.

### Scaling behavior

Based on the above analysis, the groundstate wavefunction can be expressed as 
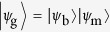
, with energy *E*_g_ = *E*_b_ + *E*_m_, where 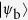 is the lower band eigenstate with energy *E*_b_, and


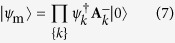


is the midgap state with energy 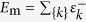, where {*k*} denotes the set of *k* within the region 2*π*/3 < *k* < 4*π*/3. In the thermodynamic limit, the band state is independent of *η*, while the midgap state is dependent of *η*. To characterize the quantum phase transition induced by *η*, we look at the second-order derivatives of the groundstate energy


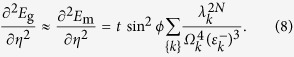


Obviously, the property of ∂^2^*E*_g_/∂*η*^2^ depends on the behavior of 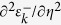. From the exact expression of 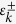 in Eq. (5), we note that the gap between 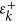 and 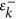 has a minimum


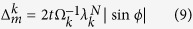


at 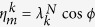, which is obtained from 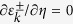. In addition, 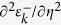 reaches the maximum


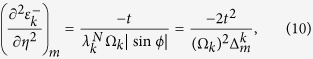


at the same point 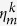. It is found that, quantities 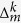, 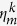, and 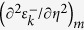 all exhibit scaling behavior. These facts should result in the scaling behavior of ∂^2^*E*_g_/∂*η*^2^. We would like to point out that, the above analysis is not applicable to the situation of *k* = *π*. In this case, we have *λ*_*k*_ = 0, which reduces the SSH ring to a trivial case. On the other hand, in the case of *ϕ* → 0, the avoided level-crossing between 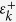 and 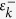 becomes a level-crossing in a finite system. The second-order QPT becomes the first-order one.

**Figure 3:**
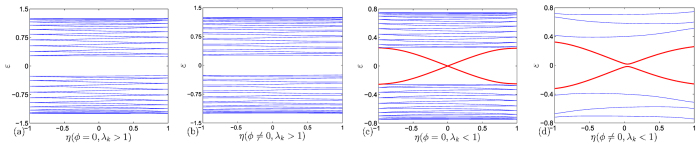
Energy spectra for the Hamiltonian in Eq. (3) on a lattice with *N* = 20 for (**a**), (**b**), (**c**) and *N* = 4 for (**d**), obtained by exact diagonalization. The parameters are (**a**) *ϕ* = 0, *λ*_*k*_ = 1.5; (**b**) *ϕ* = *π*/4, *λ*_*k*_ = 1.5; (**c**) *ϕ* = 0, *λ*_*k*_ = 0.5; (**d**) *ϕ* = *π*/4, *λ*_*k*_ = 0.5. We see that the zero modes do not appear in the cases of (**a**) and (**b**), no matter the flux is zero or not. In contrast, the zero modes appear as a level crossing and avoided level crossing, in the cases of (**c**) and (**d**), respectively. In the case of (**d**), we take a small *N* in order to demonstrate the avoided level-crossing clearly.

**Figure 4:**
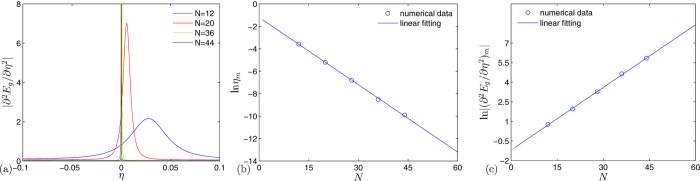
The characteristics of second-order QPT for the present system in Eq. (1). (**a**) Plots of ∂^2^*E*_g_/∂*η*^2^ as a function of *η* for different values of *N*, (**b**) the scaling law of pseudo critical point *η*_*m*_ as a function of *N*, (**c**) the scaling law of the (∂^2^*E*_g_/∂*η*^2^)_*m*_ as a function of *N*. The parameters for all plots are *M* = 7 and *ϕ* = *π*/4.

To demonstrate the origin of the critical behavior occurs in *H*_*k*_, we plot several typical band structures for *H*_*k*_ as functions of *η* in Fig. 3. It shows that in the case of |*λ*_*k*_| > 1, the band structures are unchanged when the boundary condition changes no matter the flux presents or not. In the case of |*λ*_*k*_| < 1, the main band structures are still unchanged when the boundary condition changes. There are two midgap levels emerge from the upper and lower bands, respectively. The appearance of midgap levels does not depend on the flux. However, the key feature is that the flux can lead to a level-crossing. The flux takes the role of quantum fluctuation, driving the second-order QPT. We also plot the ∂^2^*E*_g_/∂*η*^2^ as functions of *η* and *N* for nonzero *ϕ* in Fig. 4. It shows that ∂^2^*E*_g_/∂*η*^2^ has a maximum (∂^2^*E*_g_/∂*η*^2^)_*m*_ at a pseudo critical point *η*_*m*_. This predicts that, in large *N* limit, (∂^2^*E*_g_/∂*η*^2^)_*m*_ diverges at zero *η*_*m*_. The plots of *η*_*m*_ and (∂^2^*E*_g_/∂*η*^2^)_*m*_ as functions of *N* indicate the scaling law, exhibiting the second-order QPT behavior. The linear fitting allows us to estimate the scaling functions as the from


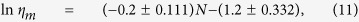



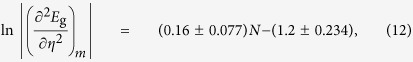


which are in good accordance with the numerical results.

Another way of looking at QPTs from the quantum information point of view is ground-state fidelity^11^,^12^. For our case, we focus on the midgap-state fidelity, which is defined as


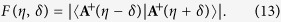


A straightforward derivation results in


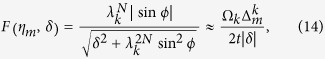


which also exhibits scaling law.

From above analytical and numerical analysis, we conclude that the real-space topology can induce a QPT. Key features of such kind of phase transition are that it is induced by a local parameter *η*, which is similar to the impurity induced QPT^13^. We would also like to point out that the flux is crucial for the transition. In the case of zero flux, the quantum transition reduces to the first-order transition. The parameter *η* drives the transition from one edge state to another through the quantum fluctuation of the flux. Another point we want to address is that the contexture of the toroid, honeycomb lattice, is also crucial for the QPT. We demonstrate this by the following system.

**Figure 5:**
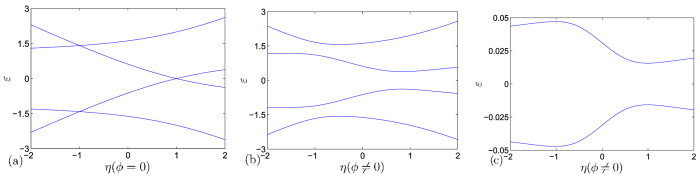
Energy spectra for the Hamiltonian in Eq. (23) on a lattice with *N* = 4 for (**a**), (**b**), and *N* = 100 for (**c**), which only shows the bottom energy level of the upper band and the top energy level of the lower band for simplicity, obtained by exact diagonalization. The parameters are (**a**) *ϕ* = 0; (**b**) *ϕ* = *π*/4; (**c**) *ϕ* = *π*/4. Here, we set *λ*_2*k*_ = 0 for all figures because it is just an on-site potential and has no influence on the band structure. We see that the zero modes appear as a level crossing in the case of (**a**) when no flux. In contrast, in case of (**b**), it seems like an avoided level-crossing happens when flux presents. But in the case of (**c**), we demonstrate that there is still a gap between the upper and lower bands even if *N* is sufficiently large, which means there is no avoided level-crossing whether the flux presents or not.

In contrast to the graphene tube, we consider a system of noninteracting particles in a square lattice, subjected to a magnetic flux *ϕ*. The tight-binding model for this system can be described by the Hamiltonian


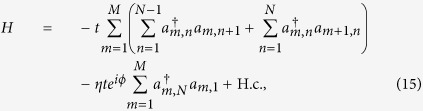


which obeys the periodic boundary conditions, *a*_*M* + 1, *n*_ = *a*_1,*n*_. From the Methods, there is no QPT for the corresponding real-space topological change in the square lattice system. To show this point more clearly, we plot several typical band structures for *H*_*k*_ described in Eq. (23) as functions of *η* in Fig. 5. It shows that in the case of *ϕ* = 0, the band structures are always level-crossing, even in the small size of the system. In the case of *ϕ* ≠ 0, the level-crossing has been broken and a gap appears between upper and lower bands, even in the very large size of the system. This means that there is no avoided level-crossing whether flux presents or not. These two examples indicate that the occurrence of a real-space induced QPT strongly depends on the contexture of the system. It also reveals a fact that the groundstate property must be tightly connected to the topology of the system in which a real-space induced QPT can happen.

## Discussion

In this work, we have demonstrated the existence of the QPT induced by the real-space topology of the system. In contrast to the conventional QPT, which is driven by a global physical parameter, such a QPT is induced by a varying local parameter. Nevertheless, the characteristics of second-order QPT, such as scaling behaviors of the second-order derivatives of groundstate energy, pseudo critical point, and the fidelity of the groundstate wavefunction, are all obtained. This finding reveals the connection between the QPT and the real-space topology, which will motivate further investigation.

## Methods

### The approximate solution of a Peierls ring with the *k*-dependent hopping integral *λ*_*k*_ = 2cos(*k*/2)

We write down the Hamiltonian (3) in the basis 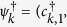 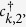 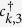, ..., 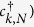 and see that


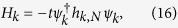


where *h*_*k,N*_ represents a *N* × *N* matrix and contains two parts, 
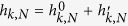
. Here two *N* × *N* matrices are


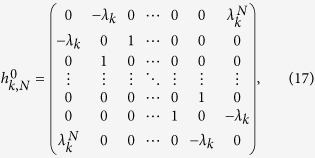


and


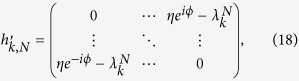


respectively. It is hard to get the explicit eigenfunctions of matrix *h*_*k,N*_. Fortunately, we have two eigenvectors with zero eigenvalue, i.e., 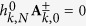, where


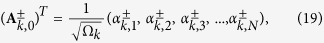


with 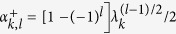, 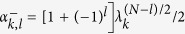 and 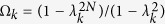  ≈  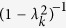. We see that in large *N* limit, *h*_*k,N*_ depicts an open chains: (i) |*λ*_*k*_| < 1, *η*_*k*_ vanishes, a *N*-site ring becomes a *N*-site chain; (ii) |*λ*_*k*_| > 1, *η*_*k*_ tends to infinity, a *N*-site ring reduces to a (*N* − 2)-site chain and a 2-site separated dimer with eigenvalues out of the bands. In both two cases, there are always two zero-mode states, in which the particle probability locates around the junction, which are so-called edge states. The solutions of both cases accord with each other. The solution for *h*_*k,N*_ with finite *N* is the basement of the scaling behavior for the geometric topological transition. Since two zero modes 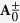 are at midgap, *h*_*k,N*_ can be regarded as a perturbation for small *η*. The degenerate perturbation method gives the


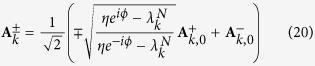


with eigenvalues


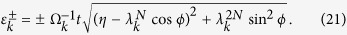


### The exact solution of the square lattice system

From the Hamiltonian (15), which obeys the periodic boundary conditions, *a*_*M* + 1,*n*_ = *a*_1,*n*_. The geometry of this system is schematically illustrated in Fig. 2(b,d). We employ the Fourier transformation


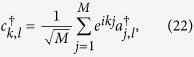


to rewrite the Hamiltonian, where *k* = 2*πm/M, m* ∈ [1, *M*]. The Hamiltonian can be still expressed as *H* = ∑_*k*_*H*_*k*_, where


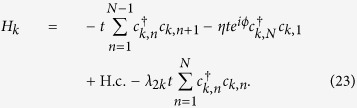


Together with [*H*_*k*_, *H*_*k*′_] = 0 and *λ*_2*k*_ = 2cos *k*, we find that *H* is a combination of *M* independent rings with the *k*-dependent on-site potential −*λ*_2*k*_*t*. The spectra of *H*_*k*_ with *η* = 1 and 0 are


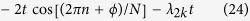


and


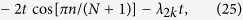


respectively, where *n* ∈ [1, *N*]. It indicates that the band structure is unchanged as the boundary condition changes. And several typical band structures for Eq. (23) have been plotted as functions of *η* in Fig. 5 to show this point clearly.

## Additional Information

**How to cite this article**: Li, C. *et al*. Quantum phase transition induced by real-space topology. *Sci. Rep.* **6**, 39416; doi: 10.1038/srep39416 (2016).

**Publisher's note:** Springer Nature remains neutral with regard to jurisdictional claims in published maps and institutional affiliations.

## Figures and Tables

**Figure 1:**
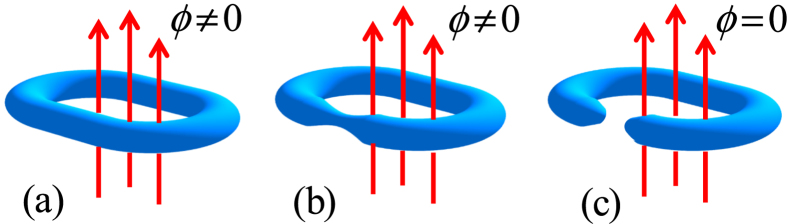
Schematic illustration of the aim of the present work. It arises from the fact that the effect of a magnetic field on an object depends on its real-space topology. We consider three cases: (**a**) A perfect torus, (**b**) a cut torus, (**c**) a broken torus. Objects in (**a**) and (**b**) have the same real-space topology, while (**c**) is topologically equivalent to a bar, with different real-space topology. Charged particles in systems (**a**) and (**b**) feel the same flux, while (**c**) cannot feel the existence of the field. The switch of the topology is equivalent to the sudden removal of the applied magnetic field, which may introduce a sudden change of the quantum state at zero temperature.
